# Emergency politics, mass sentiment and the EU during Covid

**DOI:** 10.1057/s41295-023-00330-y

**Published:** 2023-02-06

**Authors:** Chendi Wang, Abel Bojar, Ioana-Elena Oana, Zbigniew Truchlewski

**Affiliations:** 1 Department of Political Science and Public Administration, Vrije Universiteit Amsterdam, De Boelelaan 1105, 1081 HV Amsterdam, The Netherlands; 2 Department of Social and Political Sciences, European University Institute, Via dei Roccettini 9, S. Domenico di Fiesole, 50014 Florence, Italy; 3 School of Economics and Political Science, European Institute, Houghton Street, London, WC2A 2AE UK; 4 21 Research Center, Szőlő Street 88. 2./10a., 1032 Budapest, Hungary; 5 Robert Schuman Centre for Advanced Studies, European University Institute, Via dei Roccettini 9, S. Domenico di Fiesole, 50014 Florence, Italy; 6 Global Governance Centre, Geneva Graduate Institute, Chem. Eugène-Rigot 2, 1202 Geneva, Switzerland

**Keywords:** Emergency politics, European Union, Public opinion, Social media, Policy process analysis

## Abstract

During crises, do emergency politics impair the EU polity by alienating Europeans? Recent literature suggests that executive decisions in hard times can spur negative European sentiment, increase polarisation in the public and thus create more problems than solutions. The Covid-19 pandemic offers an ideal opportunity to study this important issue. However, studying mass sentiment towards the EU is mostly constrained by imperfect survey data. We tackle this challenge with an empirical strategy that combines two original data sources: first, we use policy process analysis to identify key EU decisions; second, we leverage Twitter data to measure sentiment. As a result, we can study whether key EU decisions impacted EU sentiment and whether this impact is conditional on the level of EU competence, prior politicisation and problem pressure. We find that EU decisions impact EU sentiment positively and do not polarise it (even among highly politicised decisions). Low prior politicisation and healthcare-related decisions increase the positive impact of EU actions. There is thus no punishment of the EU for acting outside its remit. Our findings have important implications for the politics of polity maintenance in the EU.

**Supplementary Information:**

The online version contains supplementary material available at 10.1057/s41295-023-00330-y.

## Introduction

The European Union (EU) has faced its fair share of crises in the last two decades. It had to react fast and decisively to highly uncertain and conflictual situations. These stress tests of the “supply side” of European politics in times of emergency have been analysed extensively by a nascent literature on crisis and emergency politics in the EU (see Bojar and Kriesi 2021; Ferrera et al. 2021; Truchlewski et al. 2021, for recent examples on Covid).

The emergency politics literature (Rhinard 2019; White 2020; Heupel et al. 2021; Kreuder-Sonnen 2019) argues that the EU has succumbed to a “crisisification”, i.e. the tendency of European executives to exploit crisis politics to circumvent democratic debate and impose policy solutions on the European public. The emergency politics literature harks back to earlier heated debates on the EU’s democratic deficit (Follesdal and Hix 2006) and thus to deeper issues that underpin European politics and crisis management.

This literature is, however, mostly theoretically focused on the “supply side” of EU politics. By contrast, we know considerably less empirically about how the European public reacts to emergency politics. This is a key question because the way the EU manages a crisis most likely impacts how citizens view the EU, which feeds back into European politics later. Do EU crisis politics weaken or strengthen its legitimacy? Under what conditions and in which issue areas are we most likely to observe such an impact? Evidence from previous crises suggests that the EU’s emergency politics may be poorly perceived even when they are highly effective or actions of last resort. For instance, Jones (2009) documents how the Euro Area crisis led to a loss of legitimacy in EU institutions.

Granted, there are structural reasons for this little amount of evidence on the “demand side” of emergency politics. First, the emergency politics literature was first conceived in the realm of political philosophy (Agamben 2005; Kalyvas 2008; Honig 2009) and is only now being operationalised in the social sciences (Rauh 2021; Truchlewski et al. 2021). As a result, our study is exploratory in nature rather than a hard test of the theory. Second, surveys do not include questions, strictly speaking, on the perceptions of emergency politics by the general public (although see Ganderson, Schelkle and Truchlewski this issue, for an exception). Additionally, surveys are rarely fielded precisely before and after emergency politics. Even if they were, they would be fielded long after the events and would therefore be prone to hindsight bias. It is therefore hard to gauge the impact of emergency politics on EU public opinion.

The contributions of this paper are to move the discussion on emergency politics from a theoretical to an empirical ground and to tackle this challenge by combining two original data sources. We exploit a novel Twitter dataset to track the real-time reaction of the public to the announcement of key decisions made by European policymakers which we measure with another innovative dataset collected through *Policy Process Analysis* (PPA, see Bojar et al. 2021, for an overview). We aim to understand whether key decisions during the first wave of the Covid pandemic increased pro-EU sentiment; whether different types of decisions impacted mass sentiment towards the EU differently; whether shifts in mass sentiment were sensitive to prior policy politicisation; and whether these shifts vary by context like the underlying problem pressure.

Next, this article reviews the literature and formulates hypotheses on the mechanisms leading to different levels of EU sentiment during the Covid-19 pandemic. We expect first that Covid-related EU decisions create positive reaction by the public. Second, such reactions are conditional on several factors: whether EU decisions were consensual (positive reaction of mass sentiment) or conflictual (negative reaction); whether EU decisions were “business as usual” or whether they expanded into new policy fields, thereby signalling the EU’s resolve to deal with the crisis (positive effect on mass sentiment); and finally whether problem pressure made the decision more visible (e.g. the higher the infection/mortality curve, the more visible the EU’s actions). The third and fourth parts describe the data and present our findings. The fifth part concludes by discussing the implications of our findings.

## Emergency politics, mass sentiment and the EU during Covid

During the first wave of the Covid-19 pandemic, the salience and executive dominance that characterised crisis management were truly extraordinary, especially if compared to past crises (Bojar and Kriesi 2021). Governments reacted decidedly in a many policy domains, from border closures, to sanitary rules, exports and macroeconomic policies. Under the widespread use of emergency powers on the national level and an unprecedented level of policymaking activity at the EU-level including policy domains that traditionally fall outside the EU’s competence, such as the coordination of procurement of healthcare equipment, purchase agreements with producers and distribution of vaccines, it is no exaggeration to speak of a paradigmatic case of emergency politics. Emergency politics is characterised by a shift from parallel debates on multiple domains by lower levels of policymaking to the serial processing of “macro-politics” at the highest level (Baumgartner et al. 2018). Simultaneously, public opinion becomes concentrated on a single-issue area resulting in an extraordinary level of accountability of policymakers who are perceived as single-handedly responsible for addressing the crisis situation.

The stakes of understanding how the EU public reacts to emergency politics are quite high. The way the EU managed the Covid crisis could spur negative sentiment and further polarisation in the public, especially as the EU is particularly vulnerable to politicisation (Oana et al., this issue). This link has important consequences for the governability of the EU. For instance, in the economic realm, if an institution like the ECB takes decisions that polarise market actors and create negative sentiment among them, it risks undermining the efficiency of its own policy instruments. If the ECB cannot shape market expectations, monetary policy loses traction. Likewise, if the EU’s institutions undermine mass sentiment, crisis resolution policies will be less efficient. Worse, they can increase polarisation and negative perceptions of the EU: policy solutions create their own political problems, and the cure becomes worse than the disease. A case in point is the refugee crisis (Kriesi et al. forthcoming): the migrant reallocation scheme created many tensions within the EU and opened the door for the exploitation of the crisis in domestic politics in countries like Hungary and Poland—thereby likely contributing to the spread of populism. More recently during Covid, travel rules and vaccination passes have arguably created similar backlashes.

Such an accountability mechanism does not necessarily imply a direct link between the severity of the pandemic (e.g. infections, deaths) and diffuse sentiment for the EU. What it does imply is that compared to normal times, citizens’ attention is likely to be anchored to a single-issue (or limited set of issues) domain. The set of actors that seeks solutions to this domain is considerably more limited and concentrated in executive institutions. For instance, parliamentary debates and extra-parliamentary initiatives of opposition parties and civil society groups are likely to get scant attention, while EU leaders, heads of governments, issue-specific ministers and task forces are likely to dominate the news. Consequently, emergency decisions taken by national and EU-level executives are likely to become subject to a higher-than-usual public scrutiny, with discernible swings in aggregate-level sentiment for the EU following key moments of the policy debate. As a baseline expectation, we anticipate that key EU-level decisions dealing with the Covid-19 pandemic lead to an increase in positive EU sentiment among the general public.

Conversely, however, during emergency politics, the EU can be trapped in “executive politics” which concentrates blame—as opposed to more diffuse modes of policymaking where blame attribution is more tricky (Weaver 1986). This is especially problematic for the EU as it has limited competencies in many domains that required coordination during the pandemic and where the public expected a solution. Health policy is such a case. By definition, acting in a policy domain where the EU has little competence cannot result in immediate solutions, whereas emergency politics are a promise to deliver swiftly at the cost of bypassing (momentarily) set rules. The EU’s decisions during the pandemic may thus trigger negative sentiment among the EU public due to lowest common denominator solutions and “failing forward” (Jones et al. 2016).

### **H1a**

Key EU decisions on the Covid-19 pandemic lead to higher positive mass sentiment towards the EU among the general public.

However, this expectation needs to be set against the heated debates on the EU’s involvement in past crises that heavily polarised public opinion on its role in crisis management (Goldberg et al. 2020; Di Mauro and Memoli 2021). While the EU’s involvement in crafting joint solutions to address the consequences of the pandemic may be generally welcomed, it may also reinforce the views of a dissenting minority that perceives this involvement as yet another assault on national sovereignty. Together with rising average positive EU sentiment, we thus also expect sentiment polarisation on the issue.

### **H1b**

Key EU decisions on the Covid-19 pandemic polarise mass sentiment towards the EU among the general public.

EU-level decisions are also likely to be preceded by mediatised debates serving as elite cues to the public. In their legitimation efforts, EU authorities seek to project consensus while national-level policymakers, engaged in two-level games (Putnam 1988) between their national publics and the EU, may have an incentive to dissent. Conversely, EU officials might voice their disagreement vis-a-vis national-level politicians when the latter seek unilateral solutions or contravene EU rules. The intensity of such bottom-up or top-down disagreements is likely to impact the EU’s perceived legitimacy. When citizens see that decisions result from consensus, they are less likely to question their legitimacy because countervailing arguments that might otherwise mobilise eurosceptic citizens will be absent. When, instead, high levels of politicisation precede decisions, opinions are more likely to be polarised, and the boost to positive levels of EU sentiment is likely to be smaller. Our second expectation thus links mass-level sentiment for the EU as well as its variance to the policy debate that preceded the key policy responses to Covid-19. Specifically, we expect that decisions preceded by intense debates dampen the positive impact of policymaking on the level of positive EU sentiment and increase its impact on sentiment polarisation.

### **H2a**

Policy decisions preceded by high levels of politicisation lead to smaller increases in average levels of positive EU sentiment compared to decisions preceded by low levels of politicisation.

### **H2b**

Policy decisions preceded by high levels of politicisation lead to larger increases in the variance of EU sentiment compared to policy decisions preceded by low levels of politicisation.

Another key characteristic of the policy decisions on Covid-19 relates to the issue area. Crucially, Covid-19 was a twin crisis unfolding in two domains of policymaking with their own sub-domains: healthcare and the economy. In healthcare, debates revolved around the supply and coordination of medical equipment—mostly masks and ventilators—vaccine purchases and distributions, and the coordination of border controls. Though the latter is not strictly speaking a health policy decision, their main purpose was to flatten the infection curve. On the economic front, debates revolved around monetary policy interventions, fiscal initiatives, single market rules, and business support measures. Whether and to what extent the EU had core competencies in these different issue areas can be consequential for the EU’s legitimacy. We expect that when the EU took a proactive approach in issue areas where its initial competencies were limited, it served as a more powerful signal to citizens that it rose to the exceptional challenge of the crisis. Paradoxically, therefore, by stepping beyond its institutional constraints in unfamiliar territories like healthcare, we expect that the EU has managed to induce a larger shift in positive sentiment compared to policy decisions taken within its core competences (e.g. monetary policy).

### **H3**

 The effect of policy decisions on mass sentiment is more positive when these decisions were taken outside the core competencies of the EU compared to issue areas that lie within the EU’s core competencies.

Finally, though the crisis has been characterised by an extraordinary level of public salience, public attention to EU-level policymaking is still likely to vary with the severity and the intensity of the crisis. Hence, we expect that in periods of increasing and/or high problem pressure, the general public holds decision-makers in general and the EU more accountable than in periods of relative calm. Increases in positive EU sentiment should thus be the largest during the peaks of the EU-wide infection curve when the severe problem pressure concentrates the issue attention of most citizens, who expect policy solutions to the crisis.

### **H4**

The effect of policy decisions on positive mass sentiment is larger in periods of high problem pressure compared to periods of moderate/low problem pressure.

## Data and methods

### Using Twitter data to measure EU sentiment

Social media data are increasingly being used to understand political phenomena, for instance to gauge issue attention (Barberá et al. 2019), public opinions on elections (Tumasjan et al. 2010) or presidential job approval (O’Connor et al. 2010). Twitter data have also been used to analyse general sentiment regarding the Covid-19 pandemic (Okango and Mwambi 2022) or to quantify the rise of vaccine opposition (Bonnevie et al. 2021). The use of social media for measuring public sentiment for the EU comes with a unique set of advantages for our study. First, compared to other data used to measure reactions to the “demand side” of EU politics such as surveys, the high granularity of social media data allows to track real-time reactions to the policies proposed, negotiated and enacted by the EU. In contrast with the most common source of EU sentiment (Eurobarometer), social media data allow to follow the daily changes in such sentiment, which enables to link the observed swings to immediately preceding events. Second, the high volume of the Twitter data we collected allows us to capture a broad geographical scope and a large number of users, a scope that is harder to achieve with surveys due to their resource-intensiveness.

However, Twitter data also have some potential drawbacks related to the representativeness of our sample. Previous research on the representativeness of Twitter data shows that users are more likely to be male and to live in urban areas compared to the general population (Barberá and Rivero 2014). This problem is exacerbated in our case by English language texts. Nevertheless, while lacking in demographic representativeness, several previous studies indicate that Twitter data are still highly correlated with political opinion poll data and are a consistent predictor of political behaviour. For example, van Klingeren et al. (2021) show a remarkable resemblance between sub-issues used on Twitter to polled public opinion data in the context of the 2016 Ukraine referendum in the Netherlands, DiGrazia et al. (2013) show that there is statistically significant association between tweets that mention a candidate for the U.S. House of Representatives and his or her subsequent electoral performance, O’Connor et al. (2010) show that surveys and Tweets correlate in sentiment word frequencies in what regards consumer confidence and political opinion, while Ceron et al. (2014) show that Twitter data tend to be well correlated with poll findings and can be used for relatively accurate predictions of election outcomes in Italy and France. The results of this stream of research increase our confidence that, while not fully representative, our data can be used as a good proxy for public sentiment towards the EU.

Given these advantages in tracking real-time reactions, we collected Tweets posted during the first wave of the Covid-19 pandemic, between the 1st of March 2020 and the 1st of August 2020 using the Academic Twitter API. Our data extraction strategy was based on obtaining the broadest possible corpus that would enable us to measure sentiment across a wide variety of users and geographical scope but retain only tweets related to the Covid-19 pandemic and EU policymaking. To achieve this, we extracted all English language Tweets posted in the period but pre-selected them on the basis of two sets of keywords and hashtags: 23 keywords related to the EU or EU institutions and 9 keywords related to Covid.^1^ In total, our corpus contains data from 197,441 users and 583,631 Tweets.^2^

Earlier studies have shown that using sentiment analysis is relatively more reliable in reflecting public opinion or election outcomes than measuring mere volumes of tweets (Ceron et al. 2014; Chung and Mustafaraj 2011). Incorporating this insight, we use sentiment analysis following a dictionary approach (while keeping information on tweet volumes as a control and a robustness test on our results). We employ a widely used dictionary for the automated coding of sentiment in textual data, the Lexicoder Sentiment Dictionary (Young and Soroka 2012). Lexicoder uses a bag-of-words approach and contains a list of ‘positive’ and ‘negative’ keywords, estimating sentiment based on the frequency of each type of keyword. In other words, we apply the dictionary to the tweet corpus in order to count the number of words that appear in the positive category and the negative category, respectively, in each tweet, and then, we take the difference between the counts. We then average the difference across all tweets on a daily basis. This results in a measure of *daily average EU sentiment*. At the same time, we also measure the dispersion of such sentiment in a measure of *daily sentiment polarisation*.

### Identifying key policy decisions and measuring contextual variables

For our main independent variables identifying key policy decisions as well as the politicisation surrounding them, the main method we rely on is Policy Process Analysis (PPA) (Bojar et al. 2021). PPA is a method for the data collection and analysis of policymaking debates relying on systematic hand-coding of media data. Similar to other methods relying on media data (e.g. PCA—political claims analysis, PEA—protest event analysis), PPA is an event-based methodology that focuses on identifying distinct actions undertaken by a variety of actors, addressing particular issues and how they unfold over time.

For studying the first wave of the Covid-19 pandemic, we manually coded articles in 7 international sources^3^ during the period running from early March 2020 to the late July EU summit when the NGEU was agreed upon. The indicators included in our PPA dataset include the actors involved in the policy debate, the forms of action they engage in (policy actions such as adoptions of policy, but also policy claims), the arena where the actions take place, and the issues addressed. On the one hand, the resulting dataset allows for the identification of key policy decisions in our period of interest together with the actors involved and the issues addressed. For this, we take an inductive approach and look only at important policy actions such as adoptions of specific policies and the issues that they address in our dataset. For the first wave, we inductively identified 19 dates on which the EU made important decisions.^4^ On the other hand, it also allows for the construction of systematic, comparative indicators. For example, our politicisation measure used as a moderator in hypothesis H2 is constructed from this dataset by taking into account a dimension of salience (the number of actions occurring in a particular policymaking episode) and a dimension of polarisation (the share of positive and negative actions of political actors in that time frame, note that this polarisation is different from the tweet polarisation which is one of the dependent variables).^5^ For H3, we distinguish between two issue areas: decisions in the public health domain which includes public health and border control issues and decisions in the economic domain which includes monetary, fiscal, and other economic issues. For H4, we operationalise problem pressure using the smoothed daily number of Covid-induced deaths in the EU.^6^

**Fig. 1:**
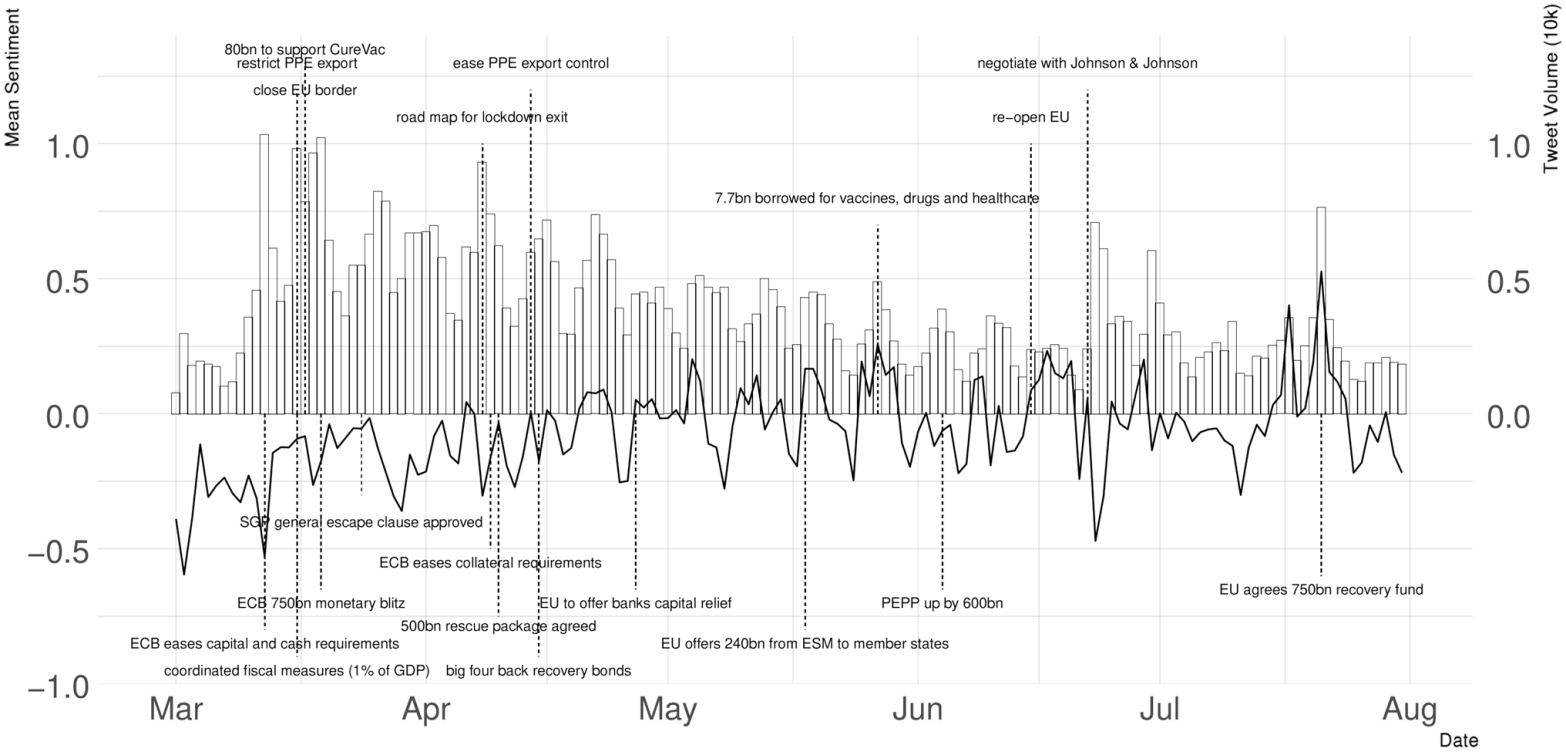
Daily tweet volume and average sentiment towards the EU in 2020

Figure 1 shows the evolution of tweets on the EU during the first wave for two indicators—(a) the daily tweet volume on the EU (in ten-thousand) and (b) the daily average EU sentiment.^7^ The bar chart reports the daily volume of tweets on the EU, and the black line describes the chronological evolution of the average sentiment towards the EU. It also marked the date of 19 decisions announced by the EU. The dashed vertical lines above the x-axis marked the decisions in the public health domain, and those below the x-axis are decisions in the economic domain. The graphs show that people tweeted a lot about the EU in March when the pandemic broke out, and then, the volume started to gradually decrease during April and May and then increased again in late June and July approaching the EU summit. We can also see that the volume of tweets seems to increase once the EU announces a decision in either the public health or the economic domain. Regarding the EU sentiment, while the average sentiment fluctuated quite a lot during the first wave of the pandemic, it was in general more negative in March and April but became more positive towards the beginning of May and stayed so throughout the summer as the infections rate dropped and lockdowns were eased. Similar to the daily volume, the average sentiment towards the EU tended to become more positive when the EU announced decisions. We can see that the most positive sentiment recorded in our dataset comes around the end of July following EU leaders' approval of the €750bn recovery fund. This is followed by the launch of the Re-open EU site and the easing of internal border restrictions in mid-June.

## Methods

After checking the stationarity of the daily series of tweet volume, sentiment mean and sentiment variance,^8^ we proceed to estimate the following dynamic model:

$$Y_{t} = \rho_{\ell } Y_{t - \ell } + \beta_{{1}} D_{t} + \beta_{{2}} P_{t} + \beta_{{3}} D_{t} P_{t} + \beta_{{4}} Z_{t} + \beta_{{5}} D_{t} Z_{t} + \varepsilon_{it}$$

for *t* = 3,*…*,*T* where *Y*_*it*_ is the daily volume, mean and variance of the sentiment series, and *D*_*t*_ represents the policy decision variables. The model also includes one lagged outcome variable for the mean sentiment, two lags for the tweet volume and three lags for the variance (polarisation) of the sentiment^9^ as well as the time-varying covariates, policy-debate politicisation *P*_*t*_ and daily deaths *Z*_*t*_. For models of average sentiment and polarisation, the daily volume is also included as predictor. Including lagged dependent variables entail dynamic model specification, and hence, the estimated coefficients of the decision dummies only describe the instantaneous impact; the total effect over time is amplified through the long-run multiplier (De Boef and Keele 2008).

## Results

We first present models that test our H1 and H2 and estimate the average (unconditional) impact of EU policy decisions of all issue domains on volume, mean sentiment and polarisation (the variance of the sentiment series) of tweets, respectively. All model specifications are dynamic, so the estimated coefficients of the decision variable merely serve to indicate the immediate response, while the total impact over time is augmented by the long-run multiplier, which is provided by the coefficient of the lagged dependent variable of the models. Models 1, 3 and 4 in Table 1 present the results of the unconditional impact of EU decisions. Model 1 confirms the finding that people tweet more about the EU when the EU announce decisions. Model 3 provides strong evidence for our H1A that the key decisions taken by the European Union have a positive impact on the mass sentiment towards the EU among the public, with around a 0.08 unit increase in terms of overall sentiment towards the EU. When taking the long-run multiplier into account, the total long-run impact is twice larger and equals a 0.16-unit increase.^10^

**Table 1:** Model of volume, mean sentiment and polarisation predicted by decisions

	Dependent variable					
	Volume	Volume	Sentiment	Polar	Sentiment	Polar
	(1)	(2)	(3)	(4)	(5)	(6)
EU decision	0.182*** (0.033)	0.082 (0.075)	0.084** (0.041)	0.036 (0.079)	0.300*** (0.085)	0.090 (0.169)
Politicise	0.199** (0.079)	0.180** (0.080)	0.085 (0.095)	− 0.170 (0.182)	0.121 (0.094)	− 0.161 (0.184)
Covid deaths	0.015*** (0.005)	0.016*** (0.005)	0.001 (0.006)	0.015 (0.013)	0.000 (0.006)	0.015 (0.013)
EU decision * politicise		0.395 (0.267)			− 0.875*** (0.301)	− 0.217 (0.599)
Volume			− 0.090 (0.095)	− 0.806*** (0.186)	− 0.057 (0.093)	− 0.799*** (0.187)
Volume_*t *− 1_	0.614*** (0.080)	0.599*** (0.081)	0.018 (0.109)	0.508** (0.220)	0.031 (0.106)	0.515** (0.221)
Volume_*t *− 2_	− 0.120 (0.074)	− 0.118 (0.073)	− 0.098 (0.085)	0.038 (0.178)	− 0.099 (0.083)	0.034 (0.179)
Sentiment_*t *− 1_			0.470*** (0.071)		0.471*** (0.070)	
Polar_*t *− 1_				0.463*** (0.080)		0.468*** (0.081)
Polar_*t *− 2_				− 0.073 (0.089)		− 0.079 (0.090)
Polar_*t *− 3_				0.287*** (0.075)		0.289*** (0.076)
Constant	0.107*** (0.023)	0.115*** (0.023)	0.007 (0.028)	0.882*** (0.246)	− 0.013 (0.028)	0.876*** (0.248)
Observations	151	151	151	150	151	150
*R*^2^	0.669	0.674	0.282	0.490	0.323	0.491
Adjusted *R*^2^	0.658	0.660	0.247	0.458	0.285	0.454
White Noise	Yes	Yes	Yes	Yes	Yes	Yes

*p < 0.1; **p < 0.05; ***p < 0.01

However, the results from Model 4 (with polarisation as the dependent variable) do not support our H1B which expects the key decisions taken by the European Union will polarise mass sentiment towards the EU. As we can see from Model 4, the coefficient of EU decision is not significant. Therefore, this result shows that key EU decisions did not polarise people. Based on this, we can conclude that during the first wave, key EU decisions did not reinforce the views of the dissenting minority.

Turning to the tests of our second block of hypotheses, we use Models 1, 3 and 4 as baselines and add interaction terms between EU decisions and politicisation. Models 2, 5 and 6 present the results. We can see in Model 2 that the interaction between EU policy decisions and politicisation is not significant, which means that the impact of EU decisions on tweet volume is not moderated by the politicisation level. However, turning to Model 5, we see that the interaction term between EU decision and politicisation is negative and significant, which offers solid evidence for our H2A. The preceding level of politicisation indeed moderates the impact of EU decisions on the mean of the sentiment series. The negative interaction term suggests that the higher level of politicisation, the less positive impact of key decisions on the sentiment towards the EU. However, the interaction term in Model 6, where polarisation is the dependent variable, is insignificant. Therefore, we find no evidence supporting H2B which expects that decisions preceded by high levels of politicisation polarise opinions.

**Fig. 2:**
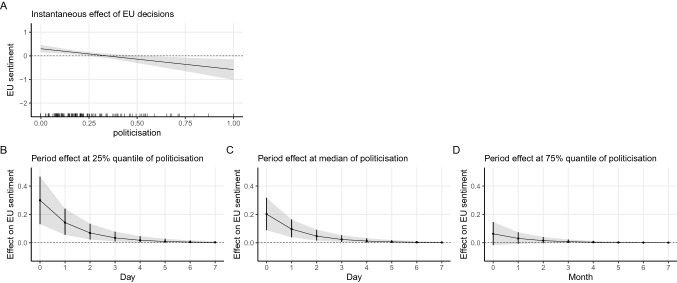
Marginal effect and simulation based on Model 5 (decision × politicisation)

We resort to marginal effect plots and dynamic simulation to illustrate the immediate and how this impact changes over time graphically.^11^ Figure 2 offers a graphical presentation of the interaction pattern. Plot A shows the instantaneous effect of EU decisions on average sentiment conditioned by the level of politicisation. Plots B, C and D illustrate how the effect of EU decisions change over time under different levels of politicisation.

As can be seen from the first plot in the first row (Plot A) of Fig. 2, in less politicised times, the marginal effect of EU decisions is positive or statistically distinguishable from zero. On the contrary, when the level of politicisation becomes higher, the estimate of the EU decisions becomes indistinguishable from zero. In cases of an extremely high level of politicisation, EU decisions even led to negative mean sentiment. Hence, H2A is well supported that EU policy decisions preceded by high levels of politicisation have a smaller positive effect on average levels of EU sentiment. When we turn to the period impact of EU decisions (Plot B-D), we get the same story where at the 25% percentile of politicisation (less politicised), the immediate increase in the mean sentiment due to an EU decision accumulates to 0.3 unit. The positive effect gradually diminishes and is gone in a week’s time, while only a 0.2 unit increases at the 50% percentile of politicisation and no impact at all if the politicisation level is high at the 75% percentile.

Proceeding to testing our H3, we now investigate the impacts of decisions in different issue domains. In particular, we introduce two intervention variables, which distinguish between decisions in the public health domain and in the economic domain to test our “core competencies” hypothesis. We first check the unconditional impact of decisions in the two domains. Models 7 and 11 in Table 2 present the results. Same as the combined indicator of EU decisions, EU decisions in both the public health and the economic domains increase the volume of tweets on the EU. Based on Model 11, however, we see no evidence supporting our H3, as the coefficients of the two interventions are insignificant. Yet, as we learned that the level of politicisation moderates the effect of decisions, we hence move to a model (Model 12) in which the two decision variables interact with the level of politicisation. Once we introduce politicisation into the model, the pattern changes. In Model 12, we see that the coefficients of the two types of decisions become significant, but the size of the effect of EU decisions in the health domain is more or less the same as in the economic domain. This means that when there is no politicisation at all, the boosts to positive mass sentiment by policy decisions in both domains are similar. This result does not support our H3 where we expect decisions outside the EU’s core competencies to have a bigger impact.

**Table 2:** Model of volume and sentiment predicted by decisions (health and economic)

	Dependent variable							
	Volume				Sentiment			
	(7)	(8)	(9)	(10)	(11)	(12)	(13)	(14)
Sentiment_*t *− 1_					0.468*** (0.072)	0.473*** (0.071)	0.471*** (0.071)	0.470*** (0.072)
Volume_*t *− 1_	0.605*** (0.078)	0.584*** (0.081)	0.611*** (0.079)	0.666*** (0.076)	0.023 (0.110)	0.044 (0.109)	0.037 (0.109)	0.019 (0.113)
Volume_*t *− 2_	− 0.119* (0.072)	− 0.112 (0.072)	− 0.121* (0.072)	− 0.058 (0.068)	− 0.102 (0.085)	− 0.112 (0.084)	− 0.102 (0.085)	− 0.105 (0.085)
Politicise	0.199** (0.077)	0.185** (0.079)	0.190** (0.078)	0.183** (0.076)	0.091 (0.096)	0.120 (0.096)	0.085 (0.095)	0.091 (0.096)
Deaths	0.016*** (0.005)	0.016*** (0.005)	0.021*** (0.006)		0.002 (0.006)	0.001 (0.006)	0.006 (0.007)	0.005 (0.007)
Volume					− 0.097 (0.098)	− 0.064 (0.097)	− 0.125 (0.097)	− 0.098 (0.106)
Health decision	0.174*** (0.047)	0.047 (0.140)		0.079 (0.061)	0.074 (0.057)	0.299* (0.162)		0.150** (0.074)
Econ decision	0.194*** (0.038)	0.106 (0.091)		0.344*** (0.055)	0.074 (0.050)	0.285*** (0.106)		0.090 (0.078)
Health decision * politicise		0.613 (0.611)				− 1.121 (0.709)		
Econ decision * politicise		0.328 (0.296)				− 0.800** (0.344)		
EU decision			0.267*** (0.045)				0.153*** (0.059)	
EU decision * deaths			− 0.031*** (0.012)				− 0.023* (0.014)	
Health*deaths				0.036** (0.017)				− 0.034 (0.021)
Econ * deaths				− 0.048*** (0.014)				− 0.008 (0.018)
Constant	0.108*** (0.022)	0.115*** (0.023)	0.100*** (0.022)	0.095*** (0.022)	0.008 (0.028)	− 0.011 (0.029)	0.005 (0.028)	0.004 (0.028)
Observations	151	151	151	151	151	151	151	151
*R*^2^	0.687	0.692	0.684	0.701	0.280	0.318	0.296	0.294
Adjusted *R*^2^	0.674	0.674	0.671	0.686	0.240	0.270	0.256	0.244
White noise	Yes	Yes	Yes	Yes	Yes	Yes	Yes	Yes

*p < 0.1; **p < 0.05; ***p < 0.01

**Fig. 3:**
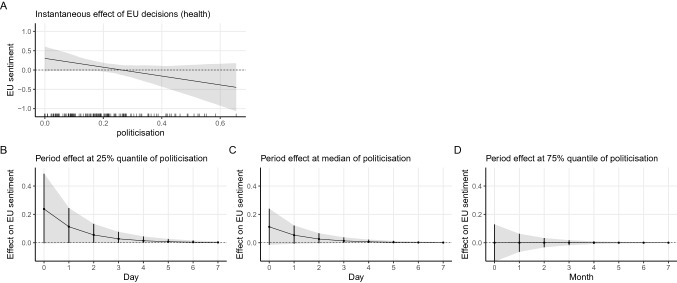
Marginal effect and simulation based on Model 12 (health × politicisation)

**Fig. 4:**
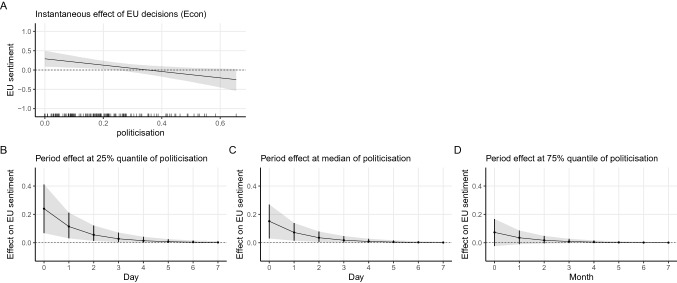
Marginal effect and simulation based on Model 12 (econ × politicisation)

In addition, the substantively large and significant interaction coefficient of EU decision in the economic domain and politicisation again offers support to our H2A. For the public health domain, the interaction of EU decisions and politicisation borders on significance. The interaction patterns are the same as the situation when all decisions are combined. Again, we illustrate the results graphically. Figures 3 and 4 show the instantaneous and period impact of decisions in the health and economic domain, respectively. The pattern is essentially the same as the one with all decisions combined shown in Fig. 2. When the level of politicisation is low, we see a boost in the sentiment towards the EU by decisions in both the public health domain and the economic domain. When the proceeding period becomes more politicised, the positive impact dwindles and even becomes negative in extreme cases. Moreover, when we compare the effect size of decisions in the two domains conditioned by politicisation, we see that decisions in public health have a similar instantaneous impact on the sentiment towards the EU, which again is against our H3. We, therefore, conclude that the boost to positive mass sentiment by policy decisions is the same irrespective of whether the issue areas lie within the EU’s core competencies.

**Fig. 5:**
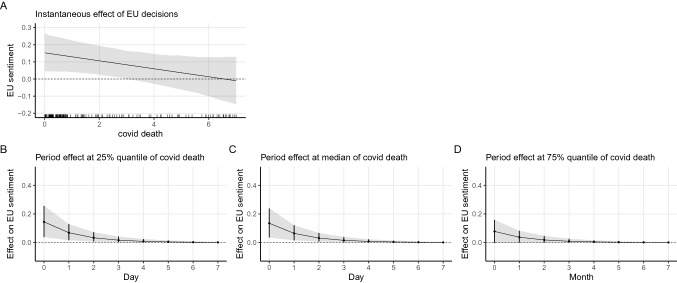
Marginal effect and simulation based on Model 13 (decision × Covid death)

Last, we turn to our H4 which expects that the positive impact on the mass sentiment of policy decisions is moderated by problem pressure. Model 9 in Table 2 presents the results on tweet volume, Model 13 shows the results on sentiment and all EU decisions, while Model 14 distinguishes between public health and economic domains. In all models, we interact our problem pressure indicator, daily Covid deaths per million (smoothed over time), with EU decisions. From Model 9, we see that there is indeed a moderating role of the Covid deaths variable. The positive effect of EU decisions on the volume of tweets on the EU becomes smaller when the problem pressure gets higher. Based on Model 13, we illustrate in Fig. 5 the interaction effect between EU decisions and problem pressure on sentiment. We can see that the positive effect of EU decisions on the sentiment towards the EU becomes smaller when the Covid-related death rate becomes higher. Therefore, our hypothesis that the boost to positive mass sentiment by policy decisions is higher in periods of high problem pressure is not supported. One of the reasons could be that we are only examining relationships between EU decisions, sentiment and problem pressure during the first wave of Covid. The results might change if a longer time span is analysed.

## Conclusion

As an experimental polity forged in crises—to paraphrase Monnet’s famous dictum—the EU runs the risk of relying too much on executives during emergency politics and thus creates the conditions for a backlash that would undermine the very polity that emergency politics try to maintain. Like other articles in this special issue, we ask whether crises can pose a threat to the EU polity by weakening legitimacy and public sentiment. The European response to the Covid-19 pandemic offers an ideal opportunity to provide some evidence on this question.

To do so, we use a research design that draws together two original databases in the context of the first wave of the pandemic. On the supply side (or at the level of policy debates), we rely on a dataset compiled via Policy Process Analysis (Bojar et al. 2021) that captures the public, mediated face of key policy decisions at the EU-level. On the demand side (or at the level of mass attitudes), we construct daily indicators of mass sentiment towards the EU based on Twitter activity. In particular, we apply a dictionary approach to gauge the daily balance between pro-EU and anti-EU tweets and use this indicator as a proxy for mass EU sentiments. To be sure, this empirical strategy is not perfect, but we do try to bring empirical substance to debates that are highly theoretical and where evidence is mostly anecdotal. In doing so, we aim to provide a baseline for future research that could build on our approach, for example, by expanding the linguistic scope of the Twitter corpus beyond English or by employing more sophisticated methods of extracting sentiment that is issue specific, such as supervised machine learning.

Our analysis, circumscribed to the first wave of the Covid-19 pandemic, is more optimistic than the emergency politics would have us think (Truchlewski et al. 2021; Alexander Shaw, Ganderson and Schelkle this issue; Ganderson, Schelkle and Truchlewski this issue). We show that key decisions during the first wave of the Covid-19 pandemic increase pro-EU sentiment and do not induce polarisation at the public level. This last result is in stark contrast to US politics, where policy decisions triggered even more political polarisation and threatened to paralyse the American political centre (Alexander Shaw, Ganderson and Schelkle this issue). Second, we distinguish between policy domains (health and economic) where the EU has different levels of competence and show that decisions in different policy domains impact mass sentiment towards the EU in a similar way. The positive impact of policy decisions in the public health domain is similar to that of economic policy decisions, indicating that when the European Union moves on from doing just “business as usual”, the EU can also enjoy increases in public sentiment. Third, our results also illustrate that one key political factor conditions the positive impact. The lower level of the prior politicisation of the policy in question, the larger boost the EU sees in terms of sentiment. Last, the problem pressure, contrary to our expectation, seems to moderate the impact of the decisions on sentiment in an opposite way. EU decisions have a larger impact when the problem pressure is low.

## Supplementary Information

Below is the link to the electronic supplementary material.

Supplementary file1 (PDF 274 kb)

## Footnotes

1 The Boolean expression we used for extracting tweets was: #EU OR #EuropeanUnion OR “EU” OR “European Union” OR “EuropeanUnion” OR “European Commission” OR #EuropeanCommission OR #EUCommission OR “European Central Bank” OR “ECB” OR #ECB OR @ECB OR @EU Commission OR “European Parliament” OR “European Council” OR #EuropeanParliament OR #EuroParl OR #EUParl OR #EuropeanCouncil OR #EUCouncil OR #EUCO OR @EUROParl OR @EUCouncil) (#corona OR #covid OR “corona” OR “covid” OR “pandemic” OR “coronavirus” OR #pandemic OR #coronavirus or “public health”.

2 The results presented here also include tweets of verified users (politicians, government official accounts etc.). As a robustness check, we also used the pool of tweets without these verified accounts, with the results remaining the same (see Appendix A3.2). Furthermore, we also discuss the geographical coverage in Appendix A3.3 and present the results based on geolocalised Twitter users, which remain the same.

3 ANSA, AP, AFP, Reuters, FT, Guardian, Euractiv, BBC.

4 In order to determine the timing of important policy decisions, we first systematically examine all policymaking debates during the sample period. We then focused on the policies in the issue areas we are interested in—public health and economic—and selected the ones that had the most extensive coverage as proxied by the large number of action counts corresponding to the same policymaking debate episode. See Appendix A1 for the full list of EU decisions.

5 The composite politicisation measure is the product of salience and polarisation as described above, weighted by the different length of the reporting newspapers to allow for the possibility that press outlets systematically differ in their coverage and standardised between 0 and 1. The first component, salience, is measured by how many actions political actors initiated during a policymaking episode. The second component, polarisation, is the multiplication of the share of positive actions among all actions in an episode and the share of negative actions. For example, if on a day during an episode there are 50% actions positive and 50% actions negative, then polarisation here is 0.25 (0.5 * 0.5). For politicisation proceeded the policy decisions, we use the 3-day moving average of this politicisation indicator with more weights attached to earlier time points. We also used the original politicisation as a robustness check which yields the same results. See Appendix A3.1.}.

6 We also used the number of new cases as a robustness check which offers the same result. See Appendix A3.1.

7 Graphical illustration of sentiment polarisation is found in Appendix A2.1.

8 Unit-root tests is found in Appendix A2.2.

9 The number of lags included is determined by comparing models with different lags based on goodness of fit criteria AIC and BIC and statistical significance of lags.

10 The long-run effect is calculated based on the following formula: *β*/(1 − *ρ*), where *β* is the instantaneous effect of EU policy decisions on mean sentiment and *ρ* is the autoregressive parameter of the mean sentiment series.

11 The corresponding confidence intervals are obtained via parametric bootstrapping of the coefficient estimates. See King et al. (2000).
