# Supplementary material for: Emergency politics, mass sentiment and the EU during Covid
Source: Comp Eur Polit. 2023 Feb 6:1–24. Online ahead of print. doi: 10.1057/s41295-023-00330-y (PMC9899657; doi:10.1057/s41295-023-00330-y)
Supplement: Supplementary file 1 — Supplementary file1 (PDF 274 kb) [file 41295_2023_330_MOESM1_ESM.pdf]

Emergency politics, mass sentiment, and the EU during COVID

- Appendix -

Contents

A1. List of EU policy decisions **A-1**

A2. Additional results **A-2**

A2.1 Sentiment polarisation over the first wave A-2

A2.2 Time series properties of tweets A-4

A3. Robustness tests **A-4**

A3.1 Alternative operationalisation A-4

A3.2 Alternative sentiment measurement: only non-verified users A-9

A3.3 Alternative sentiment measurement: geo-location specific users A-13

A1. List of EU policy decisions

Table A1. List of policy decisions

**Domain**	**Date**	**Content**
Border Control	2020 – 03 – 17	EU calls for a 30-day ban on travel to the European Union and Schengen zone
	2020 – 06 – 15	EU internal border reopened and EC has launched a site, "Re-open EU", giving information on travel, coronavirus rules, and whether hotels and beaches are open.
Public Health	2020 – 03 – 16	The European Commission said it had adopted an "authorisation scheme" to restrict sales of the safety gear outside the EU.
	2020 – 03 – 17	The European Union announced it was providing $89.4 million (80 million euros) of financial support to CureVac to scale up development and production of a vaccine against the coronavirus.
	2020 – 04 – 08	Ursula von der Leyen, commission president announce a "road map" that would guide member states on when best to end their lockdowns. The logic behind the plan was to avoid a repeat of the haphazard unilateral border closures and export bans that marked the start of the crisis.
	2020 – 04 – 14	EU to limit export control of virus protection gear to just masks.
	2020 – 05 – 27	The European Commission proposed borrowing 7.7 billion euros ($8.49 billion) on financial markets to fund extra spending on vaccines, drugs and healthcare over the next four years and reduce its dependency on foreign supplies.
	2020 – 06 – 22	The European Commission is in advanced talks with pharmaceuticals giant Johnson & Johnson to reserve or make an up-front purchase of its COVID-19 vaccine under development.
Monetary and Economic	2020 – 03 – 12	The European Central Bank decided to let euro zone banks fall short of some key capital and cash requirements as they struggle with the coronavirus outbreak.
	2020 – 03 – 19	ECB launched a €750bn monetary blitz promised to hoover up swaths of government and corporate debt to fight the downturn caused by the coronavirus.
	2020 – 04 – 09	The ECB announced "unprecedented" easing of collateral requirements in order to boost bank lending during the crisis.
	2020 – 04 – 27	EU to offer banks capital relief to help coronavirus-hit firms.
	2020 – 06 – 04	ECB announced that the pandemic emergency purchase programme (PEPP) will be increased by €600bn to a total of €1,350bn.
Fiscal	2020 – 03 – 16	The European Union finance ministers agreed on coordinated fiscal measures worth 1% of GDP to fight the economic crisis caused by the pandemic.
	2020 – 03 – 24	EU member states (ECOFIN) approved the European Commission's proposal to activate for the first time the so-called "general escape clause" that would 'pause' the adjustments member states have to do to meet their fiscal targets and allow them to spend "as much as they need".

Table A1 continued from previous page

Domain	Month of decision	Content
	2020 – 04 – 10	EU finance ministers agreed to a €500bn rescue package aimed at reducing pain across the 27-nation bloc, especially hardest-hit Italy and Spain.
	2020 – 04 – 15	EU Parliament's big four back 'recovery bonds' and green new deal.
	2020 – 05 – 18	The EU is offering up to €240bn in special credit from its rescue fund, the European Stability Mechanism (ESM), to help states deal with the economic fallout from the virus, offering zero interest rates and few conditions to its member states.
	2020 – 07 – 21	EU leaders approved a landmark stimulus package (€750bn) to fight the withering aftershocks of the coronavirus outbreak that has sunk Europe into its deepest recession in history.

A2. Additional results

A2.1 Sentiment polarisation over the first wave

Figure A1. Daily sentiment polarisation

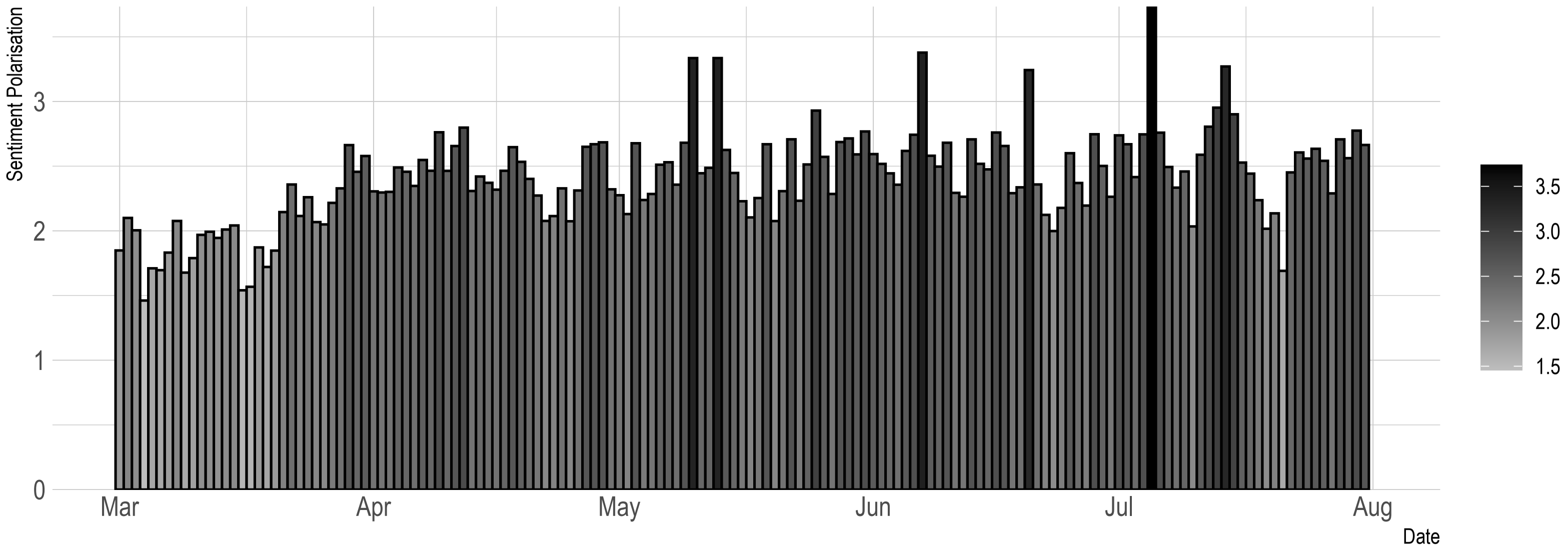

A2.2 Time series properties of tweets

The table below presents the p values for unit-root tests for tweet volume, sentiment and polarisation. Augmented Dickey Fuller test and Phillips Perron test are used. As all of the p-values are less than 0.05, the series are stationary which permits dynamic modelling via autoregressive distributed lag models.

Table A2. p-values of unit-root tests

	ADF test	Phillips-Perron test
volume	0.02	0.01
sentiment	0.01	0.01
polarisation	0.01	0.01

A3. Robustness tests

A3.1 Alternative operationalisation

We check different operationalisation strategies for politicisation and problem pressure. First, instead of a 3-day rolling average for politicisation, we use the original measurement. Second, we use daily new cases per million instead of news deaths per million. Our results in the main text hold with alternative operationalisation.

Table A3. Model of volume, mean sentiment and polarisation predicted by decisions (combined) with original politicisation

Dependent variables	volume	volume	sentiment	polar	sentiment	polar
	A1	A2	A3	A4	A5	A6
EU_decision	0.145^{***}	0.099	$0.071^{\dagger}$	0.033	0.279^{***}	0.156
	(0.032)	(0.065)	(0.041)	(0.080)	(0.075)	(0.152)
politicize(o)	0.248^{***}	0.233^{***}	0.158^{*}	0.030	0.224^{**}	0.069
	(0.051)	(0.054)	(0.067)	(0.129)	(0.068)	(0.135)
covid_deaths	0.012^{*}	0.012^{*}	0.000	0.016	−0.001	0.016
	(0.005)	(0.005)	(0.006)	(0.013)	(0.006)	(0.013)
EU_decision×politicize(o)		0.124			-0.573^{**}	−0.336
		(0.152)			(0.175)	(0.353)
volume			−0.161	-0.863^{***}	−0.140	-0.854^{***}
			(0.099)	(0.196)	(0.096)	(0.196)
volume_{t-1}	0.582^{***}	0.585^{***}	0.030	0.457^{*}	0.004	0.441^{*}
	(0.074)	(0.074)	(0.105)	(0.215)	(0.101)	(0.216)
volume_{t-2}	−0.032	−0.038	−0.050	0.038	−0.019	0.052
	(0.072)	(0.073)	(0.086)	(0.183)	(0.084)	(0.183)
sentiment_{t-1}			0.454^{***}		0.443^{***}	
			(0.070)		(0.067)	
polar_{t-1}				0.458^{***}		0.457^{***}
				(0.081)		(0.081)
polar_{t-2}				−0.079		−0.088
				(0.089)		(0.090)
polar_{t-3}				0.277^{***}		0.282^{***}
				(0.075)		(0.075)
(Intercept)	0.087^{***}	0.090^{***}	−0.001	0.934^{***}	−0.019	0.940^{***}
	(0.022)	(0.022)	(0.028)	(0.244)	(0.027)	(0.244)
R^2	0.703	0.704	0.306	0.487	0.354	0.491
Adj. R^2	0.692	0.692	0.272	0.454	0.318	0.454
Num. obs.	151	151	151	150	151	150
White Noise	Yes	Yes	Yes	Yes	Yes	Yes

$^{***}p < 0.001$; $^{**}p < 0.01$; $^{*}p < 0.05$; $^{\dagger}p < 0.1$

Table A4. Model of volume and sentiment predicted by decisions (health and economic separated) with original politicisation

Dependent variables	volume	volume	volume	volume	sentiment	sentiment	sentiment	sentiment
	A7	A8	A9	A10	A11	A12	A13	A14
EU_decision			0.230^{***}				0.147^{*}	
			(0.044)				(0.058)	
EU_decision(health)	0.142^{**}	0.081		0.054	0.064	0.382^{*}		$0.139^{\dagger}$
	(0.045)	(0.136)		(0.057)	(0.057)	(0.159)		(0.073)
EU_decision(econ)	0.160^{***}	0.108		0.312^{***}	0.062	0.261^{**}		0.094
	(0.038)	(0.075)		(0.052)	(0.049)	(0.088)		(0.077)
politicize(o)	0.237^{***}	0.221^{***}	0.245^{***}	0.249^{***}	0.161^{*}	0.221^{**}	0.166^{*}	0.166^{*}
	(0.050)	(0.054)	(0.050)	(0.048)	(0.067)	(0.068)	(0.066)	(0.067)
covid_deaths	0.013^{*}	0.013^{*}	0.018^{**}		0.001	−0.001	0.005	0.005
	(0.005)	(0.005)	(0.005)		(0.006)	(0.006)	(0.007)	(0.007)
EU_decision(health)×politicize(o)		0.164				-0.871^{*}		
		(0.342)				(0.402)		
EU_decision(econ)×politicize(o)		0.130				-0.511^{**}		
		(0.163)				(0.192)		
EU_decision×covid_deaths			-0.032^{**}				$-0.026^{\dagger}$	
			(0.011)				(0.014)	
EU_decision(health)×covid_deaths				0.032^{*}				−0.034
				(0.016)				(0.021)
EU_decision(econ)×covid_deaths				-0.050^{***}				−0.013
				(0.013)				(0.018)
volume					−0.168	−0.142	-0.204^{*}	−0.182
					(0.101)	(0.098)	(0.101)	(0.110)
volume_{t-1}	0.579^{***}	0.582^{***}	0.577^{***}	0.620^{***}	0.036	0.016	0.051	0.039
	(0.072)	(0.074)	(0.072)	(0.069)	(0.106)	(0.103)	(0.104)	(0.109)
volume_{t-2}	−0.035	−0.042	−0.033	0.022	−0.053	−0.026	−0.052	−0.056
	(0.071)	(0.072)	(0.071)	(0.066)	(0.086)	(0.085)	(0.085)	(0.086)
sentiment_{t-1}					0.452^{***}	0.441^{***}	0.453^{***}	0.453^{***}
					(0.070)	(0.068)	(0.069)	(0.070)
(Intercept)	0.088^{***}	0.092^{***}	0.079^{***}	0.074^{***}	0.000	−0.020	−0.003	−0.003
	(0.022)	(0.022)	(0.022)	(0.021)	(0.028)	(0.028)	(0.027)	(0.028)
R^2	0.716	0.718	0.718	0.738	0.304	0.358	0.322	0.320
Adj. R^2	0.704	0.702	0.707	0.725	0.265	0.312	0.284	0.271
Num. obs.	151	151	151	151	151	151	151	151
White Noise	Yes	Yes	Yes	Yes	Yes	Yes	Yes	Yes

$^{***}p < 0.001$; $^{**}p < 0.01$; $^{*}p < 0.05$; $^{\dagger}p < 0.1$

Table A5. Model of volume, mean sentiment and polarisation predicted by decisions (combined) with new cases per million

Dependent variables	volume	volume	sentiment	polar	sentiment	polar
	A15	A16	A17	A18	A19	A20
EU_decision	0.182^{***}	0.087	0.083^{*}	0.040	0.300^{***}	0.098
	(0.032)	(0.074)	(0.041)	(0.079)	(0.085)	(0.169)
politicize	0.228^{**}	0.210^{**}	0.085	−0.145	0.119	−0.136
	(0.078)	(0.079)	(0.096)	(0.186)	(0.094)	(0.188)
covid_cases	0.002^{***}	0.002^{***}	0.000	0.002	−0.000	0.002
	(0.001)	(0.001)	(0.001)	(0.002)	(0.001)	(0.002)
EU_decision×politicize		0.377			-0.877^{**}	−0.233
		(0.262)			(0.300)	(0.598)
volume			−0.086	-0.820^{***}	−0.053	-0.813^{***}
			(0.097)	(0.190)	(0.095)	(0.191)
volume_{t-1}	0.585^{***}	0.571^{***}	0.018	0.504^{*}	0.031	0.511^{*}
	(0.080)	(0.080)	(0.109)	(0.220)	(0.106)	(0.221)
volume_{t-2}	$-0.142^{\dagger}$	$-0.139^{\dagger}$	−0.093	0.033	−0.095	0.029
	(0.073)	(0.073)	(0.086)	(0.180)	(0.084)	(0.181)
sentiment_{t-1}			0.469^{***}		0.471^{***}	
			(0.072)		(0.070)	
polar_{t-1}				0.464^{***}		0.469^{***}
				(0.080)		(0.081)
polar_{t-2}				−0.074		−0.080
				(0.089)		(0.090)
polar_{t-3}				0.289^{***}		0.291^{***}
				(0.075)		(0.076)
(Intercept)	0.101^{***}	0.108^{***}	0.006	0.871^{***}	−0.014	0.865^{***}
	(0.022)	(0.022)	(0.028)	(0.243)	(0.028)	(0.244)
R^2	0.681	0.685	0.282	0.490	0.323	0.491
Adj. R^2	0.670	0.672	0.247	0.458	0.285	0.454
Num. obs.	151	151	151	150	151	150
White Noise	Yes	Yes	Yes	Yes	Yes	Yes

$^{***}p < 0.001$; $^{**}p < 0.01$; $^{*}p < 0.05$; $^{\dagger}p < 0.1$

Table A6. Model of volume and sentiment predicted by decisions (health and economic separated) with new cases per million

Dependent variables	volume	volume	volume	volume	sentiment	sentiment	sentiment	sentiment
	A21	A21	A22	A23	A24	A25	A26	A27
EU_decision			0.281^{***}				0.173^{**}	
			(0.051)				(0.066)	
EU_decision(health)	0.170^{***}	0.024		0.086	0.074	$0.299^{\dagger}$		$0.146^{\dagger}$
	(0.046)	(0.138)		(0.070)	(0.057)	(0.162)		(0.085)
EU_decision(econ)	0.197^{***}	0.125		0.361^{***}	0.074	0.285^{**}		0.147
	(0.038)	(0.090)		(0.065)	(0.050)	(0.106)		(0.090)
politicize	0.229^{**}	0.217^{**}	0.229^{**}	0.192^{*}	0.091	0.119	0.091	0.098
	(0.076)	(0.078)	(0.077)	(0.077)	(0.097)	(0.096)	(0.096)	(0.097)
covid_cases	0.002^{***}	0.002^{***}	0.003^{***}		0.000	−0.000	0.001	0.001
	(0.001)	(0.001)	(0.001)		(0.001)	(0.001)	(0.001)	(0.001)
EU_decision(health)×politicize		0.691				−1.124		
		(0.599)				(0.710)		
EU_decision(econ)×politicize		0.274				-0.803^{*}		
		(0.291)				(0.343)		
EU_decision×covid_cases			-0.004^{*}				$-0.003^{\dagger}$	
			(0.002)				(0.002)	
EU_decision(health)×covid_cases				0.003				−0.003
				(0.002)				(0.003)
EU_decision(econ)×covid_cases				-0.006^{**}				−0.003
				(0.002)				(0.003)
volume					−0.093	−0.060	−0.121	−0.124
					(0.100)	(0.099)	(0.098)	(0.106)
volume_{t-1}	0.576^{***}	0.552^{***}	0.591^{***}	0.670^{***}	0.023	0.045	0.044	0.046
	(0.078)	(0.080)	(0.078)	(0.078)	(0.110)	(0.109)	(0.109)	(0.113)
volume_{t-2}	-0.141^{*}	$-0.133^{\dagger}$	-0.154^{*}	−0.065	−0.097	−0.108	−0.108	−0.113
	(0.071)	(0.071)	(0.072)	(0.069)	(0.086)	(0.085)	(0.086)	(0.087)
sentiment_{t-1}					0.468^{***}	0.473^{***}	0.476^{***}	0.473^{***}
					(0.072)	(0.071)	(0.071)	(0.072)
(Intercept)	0.102^{***}	0.109^{***}	0.093^{***}	0.094^{***}	0.006	−0.012	0.002	0.004
	(0.022)	(0.022)	(0.022)	(0.022)	(0.028)	(0.028)	(0.028)	(0.028)
R^2	0.699	0.703	0.694	0.691	0.280	0.318	0.297	0.292
Adj. R^2	0.686	0.687	0.681	0.676	0.239	0.270	0.258	0.241
Num. obs.	151	151	151	151	151	151	151	151
White Noise	Yes	Yes	Yes	Yes	Yes	Yes	Yes	Yes

$^{***}p < 0.001$; $^{**}p < 0.01$; $^{*}p < 0.05$; $^{\dagger}p < 0.1$

A3.2 Alternative sentiment measurement: only non-verified users

In the main text, we use a sentiment variable based on all tweets that contain EU related key words. This pool of tweets include tweets from verified twitter users, i.e. those with a blue tick on their account profile. These users are usually politicians, government official accounts etc. Therefore, to test robustness of our results and purely measure the sentiment of the public, we remove the tweets from verified users and recalculate the volume, sentiment and polarisation. After removing these tweets, we now have 186,846 users and 527,517 tweets. The results based on this dataset remain essentially the same as those in the main text.

Figure A2. Daily tweet volume and average sentiment towards the EU based on non-verified users

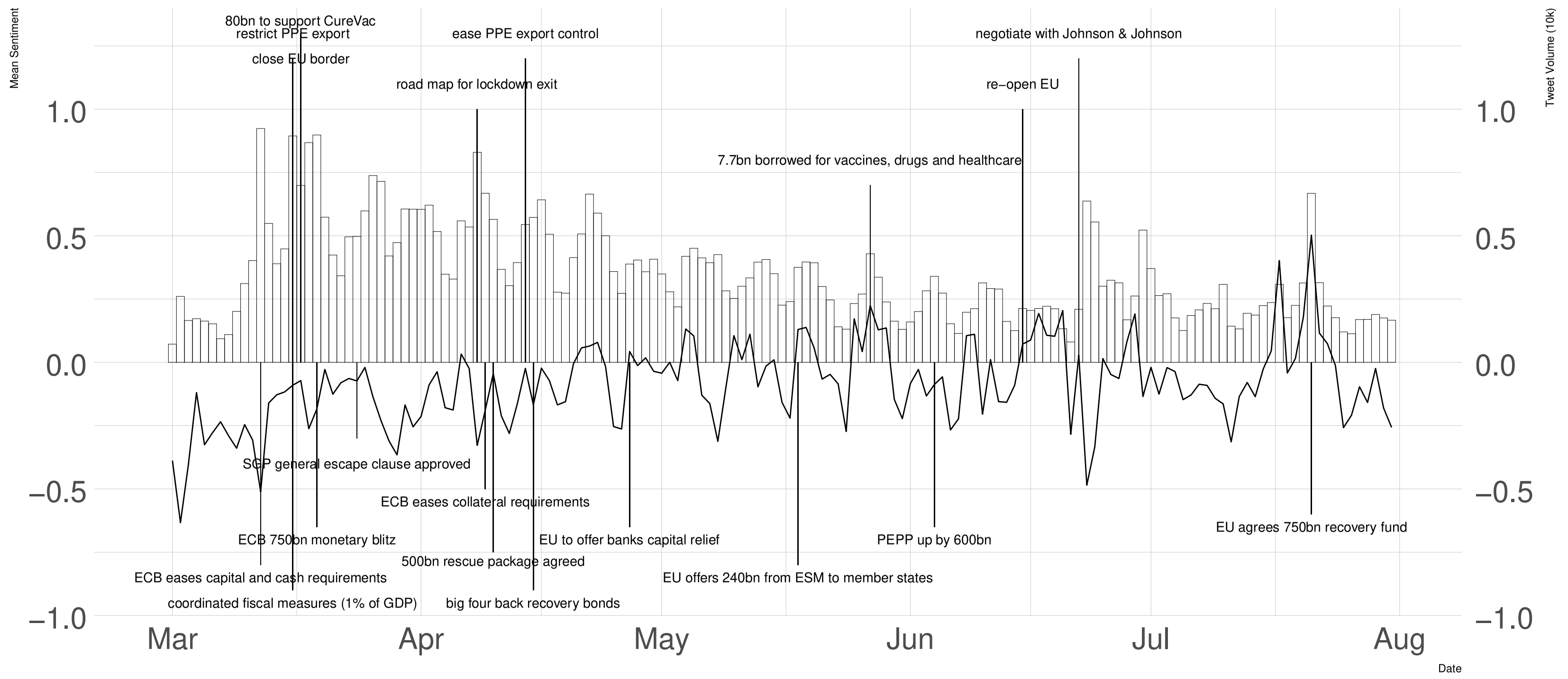

Table A7. Model of volume, mean sentiment and polarisation based on non-verified users predicted by decisions (combined)

Dependent variables	volume	volume	sentiment	polar	sentiment	polar
	A28	A29	A30	A31	A32	A33
EU_decision	0.158***	0.071	0.087*	0.016	0.290***	0.074
	(0.029)	(0.066)	(0.041)	(0.086)	(0.084)	(0.184)
politicize	0.168*	0.151*	0.086	−0.145	0.121	−0.135
	(0.069)	(0.070)	(0.094)	(0.197)	(0.093)	(0.200)
covid_deaths	0.013**	0.013**	0.001	0.013	−0.000	0.013
	(0.005)	(0.005)	(0.006)	(0.014)	(0.006)	(0.014)
EU_decision×politicize		0.340			−0.821**	−0.230
		(0.235)			(0.300)	(0.652)
volume			−0.103	−0.908***	−0.069	−0.899***
			(0.107)	(0.230)	(0.106)	(0.232)
volume_{t-1}	0.614***	0.599***	0.016	0.496†	0.031	0.503†
	(0.081)	(0.081)	(0.124)	(0.271)	(0.121)	(0.273)
volume_{t-2}	−0.093	−0.090	−0.085	0.123	−0.089	0.119
	(0.075)	(0.074)	(0.097)	(0.220)	(0.095)	(0.221)
sentiment_{t-1}			0.450***		0.451***	
			(0.072)		(0.070)	
polar_{t-1}				0.421***		0.424***
				(0.080)		(0.081)
polar_{t-2}				−0.055		−0.060
				(0.088)		(0.089)
polar_{t-3}				0.291***		0.293***
				(0.076)		(0.077)
(Intercept)	0.091***	0.097***	−0.012	0.930***	−0.031	0.923***
	(0.020)	(0.020)	(0.028)	(0.261)	(0.028)	(0.263)
R^2	0.677	0.682	0.261	0.451	0.298	0.451
Adj. R^2	0.666	0.668	0.224	0.416	0.258	0.412
Num. obs.	151	151	151	150	151	150
White Noise	Yes	Yes	Yes	Yes	Yes	Yes

$^{***}p < 0.001$; $^{**}p < 0.01$; $^{*}p < 0.05$; $^{\dagger}p < 0.1$

Table A8. Model of volume and sentiment based on non-verified users predicted by decisions (health and economic separated)

Dependent variables	volume	volume	volume	volume	sentiment	sentiment	sentiment	sentiment
	A34	A35	A36	A37	A38	A39	A40	A41
EU_decision			0.232^{***}				0.157^{**}	
			(0.040)				(0.058)	
EU_decision(health)	0.154^{***}	0.051		0.069	0.077	$0.296^{\dagger}$		0.159^{*}
	(0.041)	(0.123)		(0.053)	(0.057)	(0.162)		(0.073)
EU_decision(econ)	0.167^{***}	0.089		0.301^{***}	0.079	0.273^{*}		0.090
	(0.034)	(0.080)		(0.048)	(0.049)	(0.106)		(0.077)
politicize	0.169^{*}	0.155^{*}	0.161^{*}	0.155^{*}	0.092	0.119	0.087	0.092
	(0.068)	(0.069)	(0.068)	(0.066)	(0.094)	(0.094)	(0.093)	(0.094)
covid_deaths	0.014^{**}	0.014^{**}	0.018^{***}		0.001	0.001	0.006	0.005
	(0.005)	(0.005)	(0.005)		(0.006)	(0.006)	(0.007)	(0.007)
EU_decision(health)×politicize		0.499				−1.087		
		(0.538)				(0.708)		
EU_decision(econ)×politicize		0.293				-0.741^{*}		
		(0.261)				(0.343)		
EU_decision×covid_deaths			-0.028^{**}				$-0.024^{\dagger}$	
			(0.010)				(0.014)	
EU_decision(health)×covid_deaths				0.032^{*}				$-0.036^{\dagger}$
				(0.015)				(0.021)
EU_decision(econ)×covid_deaths				-0.043^{***}				−0.007
				(0.012)				(0.018)
volume					−0.113	−0.078	−0.143	−0.109
					(0.111)	(0.110)	(0.109)	(0.119)
volume_{t-1}	0.604^{***}	0.583^{***}	0.611^{***}	0.664^{***}	0.023	0.048	0.037	0.014
	(0.079)	(0.081)	(0.079)	(0.076)	(0.125)	(0.125)	(0.124)	(0.128)
volume_{t-2}	−0.091	−0.084	−0.094	−0.030	−0.090	−0.103	−0.089	−0.092
	(0.073)	(0.073)	(0.073)	(0.069)	(0.097)	(0.096)	(0.096)	(0.097)
sentiment_{t-1}					0.448^{***}	0.453^{***}	0.449^{***}	0.449^{***}
					(0.073)	(0.071)	(0.072)	(0.072)
(Intercept)	0.091^{***}	0.097^{***}	0.084^{***}	0.079^{***}	−0.011	−0.028	−0.014	−0.015
	(0.020)	(0.020)	(0.020)	(0.019)	(0.028)	(0.029)	(0.028)	(0.028)
R^2	0.695	0.699	0.692	0.710	0.258	0.294	0.275	0.275
Adj. R^2	0.682	0.682	0.679	0.696	0.217	0.243	0.234	0.223
Num. obs.	151	151	151	151	151	151	151	151
White Noise	Yes	Yes	Yes	Yes	Yes	Yes	Yes	Yes

$^{***}p < 0.001$; $^{**}p < 0.01$; $^{*}p < 0.05$; $^{\dagger}p < 0.1$

A3.3 Alternative sentiment measurement: geo-location specific users

When collecting Twitter data, we scrapped all English tweets that contain EU-related and COVID-specific keywords from March to August 2020. We focus on English tweets because

1. English tweets are the most prevalent ones on Twitter even for European issues.

2. Pooling measurements for cross-lingual comparison is extremely resource-intensive, and current state-of-the-art neural machine translation (NMT) methods do not handle language used on Twitter well and do not always yield reliable translations.

3. Since the conversation over EU policies during COVID is taking place on a Europe-wide scale, numerous news reports are in English, and based on our sample, we see many users who tweet in English with a location in Europe as shown in their profile.

It is optimal to filter the tweets by their tweet-specific geolocations. However, data on tweet-specific geolocations are usually not available, as most tweets do not have a geolocation tag. If we only use those tweets which have geolocation information, we will lose most of the tweets. An alternative is to use the geolocation information of the user. Most of the users have location information on their profiles, and therefore we could exclude those who are not based in Europe. One drawback of this approach is that they could be Europeans but are expatriates based in other parts of the world.

To check whether our results are robust against the sample bias, we repeat our analysis after excluding those users who have geolocations such as the US, India, and Worldwide and those who do not have geolocation information in their profiles. We ended up with a 30% reduction in our sample size. In the following tables, you can find the analysis results which are extremely similar to the ones in the main text, if not the same.

Figure A3. Daily tweet volume and average sentiment towards the EU excluding non-European users

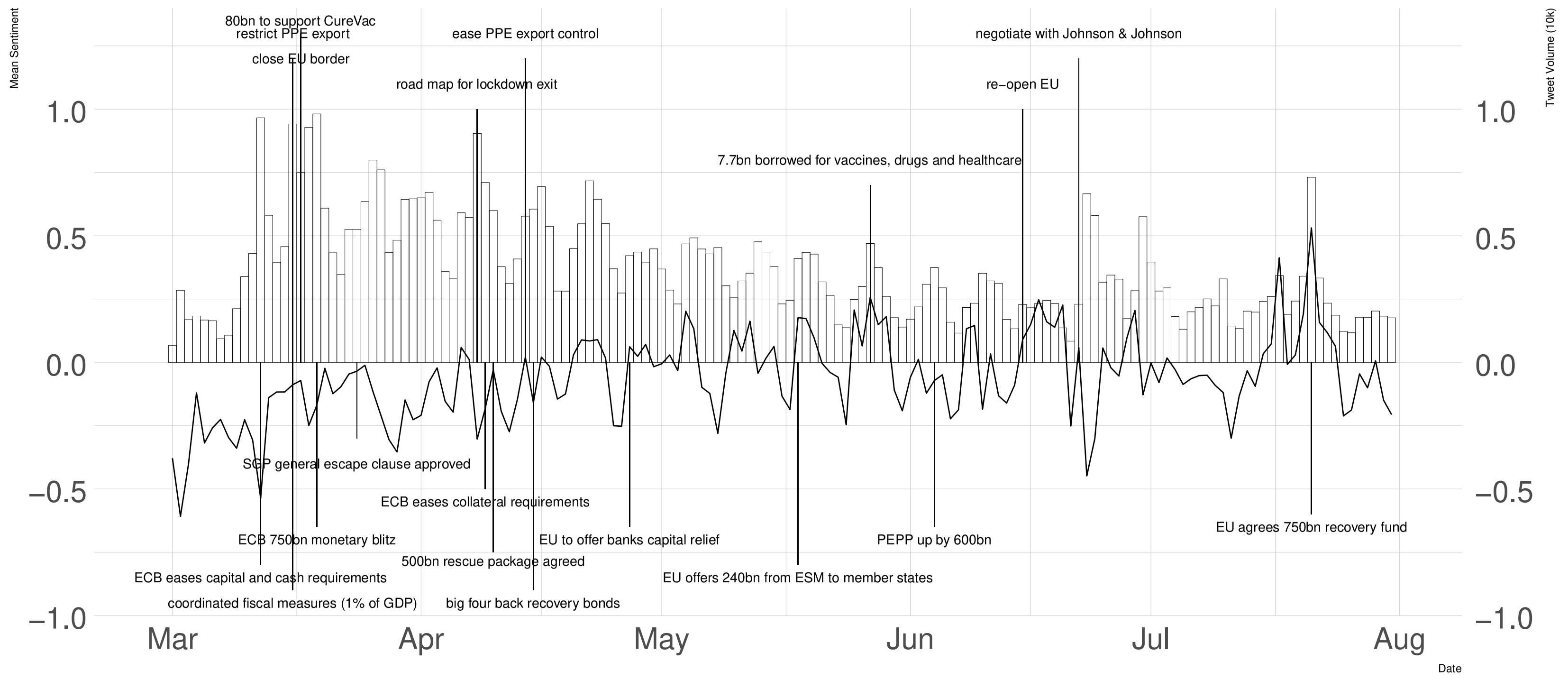

Table A9. Model of volume, mean sentiment and polarisation based on more localised tweets predicted by decisions (combined)

Dependent variables	volume	volume	sentiment	polar	sentiment	polar
	A42	A43	A44	A45	A46	A47
EU_decision	0.135^{***}	0.072	$0.083^{\dagger}$	0.081	0.300^{***}	0.098
	(0.024)	(0.056)	(0.043)	(0.076)	(0.088)	(0.163)
politicize	0.148^{*}	0.136^{*}	0.086	−0.097	0.123	−0.094
	(0.059)	(0.060)	(0.099)	(0.173)	(0.098)	(0.176)
covid_deaths	0.012^{**}	0.012^{**}	0.004	0.013	0.003	0.013
	(0.004)	(0.004)	(0.007)	(0.012)	(0.006)	(0.012)
EU_decision×politicize		0.248			-0.869^{**}	−0.068
		(0.199)			(0.311)	(0.576)
volume			$-0.224^{\dagger}$	-1.187^{***}	−0.187	-1.185^{***}
			(0.132)	(0.240)	(0.130)	(0.242)
volume_{t-1}	0.621^{***}	0.608^{***}	0.076	0.752^{**}	0.096	0.755^{*}
	(0.080)	(0.081)	(0.152)	(0.288)	(0.149)	(0.290)
volume_{t-2}	$-0.132^{\dagger}$	$-0.130^{\dagger}$	−0.156	−0.003	−0.156	−0.005
	(0.074)	(0.073)	(0.118)	(0.232)	(0.116)	(0.233)
sentiment_{t-1}			0.520^{***}		0.519^{***}	
			(0.068)		(0.067)	
polar_{t-1}				0.468^{***}		0.469^{***}
				(0.081)		(0.082)
polar_{t-2}				−0.093		−0.095
				(0.089)		(0.091)
polar_{t-3}				0.251^{**}		0.252^{**}
				(0.076)		(0.077)
(Intercept)	0.077^{***}	0.081^{***}	0.065^{*}	0.979^{***}	0.046	0.978^{***}
	(0.016)	(0.017)	(0.028)	(0.245)	(0.029)	(0.246)
R^2	0.670	0.674	0.350	0.452	0.384	0.452
Adj. R^2	0.659	0.660	0.319	0.417	0.349	0.412
Num. obs.	151	151	151	150	151	150
White Noise	Yes	Yes	Yes	Yes	Yes	Yes

$^{***}p < 0.001$; $^{**}p < 0.01$; $^{*}p < 0.05$; $^{\dagger}p < 0.1$

Table A10. Model of volume and sentiment based on more localised tweets by decisions (health and economic separated)

Dependent variables	volume	volume	volume	volume	sentiment	sentiment	sentiment	sentiment
	A48	A49	A50	A51	A52	A53	A54	A55
EU_decision			0.199^{***}				0.152^{*}	
			(0.034)				(0.061)	
EU_decision(health)	0.132^{***}	0.032		0.060	0.070	$0.320^{\dagger}$		$0.147^{\dagger}$
	(0.035)	(0.105)		(0.045)	(0.060)	(0.168)		(0.077)
EU_decision(econ)	0.181^{***}	0.108		0.326^{***}	0.070	0.289^{**}		0.078
	0.140^{***}	0.090		0.254^{***}	0.076	0.281^{*}		0.089
	(0.029)	(0.068)		(0.041)	(0.051)	(0.110)		(0.081)
	(0.074)	(0.076)	(0.075)	(0.073)	(0.098)	(0.097)	(0.097)	(0.097)
politicize	0.149^{*}	0.141^{*}	0.141^{*}	0.136^{*}	0.090	0.119	0.086	0.090
	(0.058)	(0.059)	(0.058)	(0.057)	(0.100)	(0.099)	(0.098)	(0.099)
covid_deaths	0.012^{**}	0.012^{**}	0.016^{***}		0.005	0.004	0.009	0.008
	(0.004)	(0.004)	(0.004)		(0.007)	(0.006)	(0.007)	(0.007)
EU_decision(health)×politicize		0.480				$-1.236^{\dagger}$		
		(0.456)				(0.735)		
EU_decision(econ)×politicize		0.192				-0.778^{*}		
		(0.221)				(0.356)		
EU_decision×covid_deaths			-0.024^{**}				-0.023	
			(0.009)				(0.015)	
EU_decision(health)×covid_deaths				0.028^{*}				-0.035
				(0.013)				(0.022)
EU_decision(econ)×covid_deaths				-0.036^{***}				-0.007
				(0.010)				(0.019)
volume					$-0.233^{\dagger}$	-0.191	-0.271^{*}	-0.230
					(0.136)	(0.135)	(0.135)	(0.147)
volume_{t-1}	0.613^{***}	0.590^{***}	0.619^{***}	0.673^{***}	0.083	0.118	0.104	0.077
	(0.079)	(0.081)	(0.079)	(0.076)	(0.154)	(0.153)	(0.153)	(0.158)
volume_{t-2}	$-0.131^{\dagger}$	$-0.123^{\dagger}$	$-0.133^{\dagger}$	-0.065	-0.162	-0.176	-0.164	-0.167
	(0.072)	(0.072)	(0.072)	(0.068)	(0.119)	(0.117)	(0.118)	(0.119)
sentiment_{t-1}					0.519^{***}	0.523^{***}	0.520^{***}	0.521^{***}
					(0.069)	(0.068)	(0.068)	(0.069)
(Intercept)	0.077^{***}	0.082^{***}	0.071^{***}	0.068^{***}	0.066^{*}	0.047	0.062^{*}	0.062^{*}
	(0.016)	(0.017)	(0.016)	(0.016)	(0.029)	(0.029)	(0.028)	(0.029)
R^2	0.687	0.691	0.686	0.701	0.348	0.382	0.362	0.361
Adj. R^2	0.674	0.674	0.673	0.687	0.312	0.338	0.326	0.315
Num. obs.	151	151	151	151	151	151	151	151
White Noise	Yes	Yes	Yes	Yes	Yes	Yes	Yes	Yes

$^{***}p < 0.001$; $^{**}p < 0.01$; $^{*}p < 0.05$; $^{\dagger}p < 0.1$
